# Context-Aware Multi-Agent Architecture for Wildfire Insights

**DOI:** 10.3390/s26031070

**Published:** 2026-02-06

**Authors:** Ashen Sandeep, Sithum Jayarathna, Sunera Sandaruwan, Venura Samarappuli, Dulani Meedeniya, Charith Perera

**Affiliations:** 1 Department of Computer Science and Engineering, University of Moratuwa, Moratuwa 10400, Sri Lanka; ashensandeep.21@cse.mrt.ac.lk (A.S.); sithum.21@cse.mrt.ac.lk (S.J.); sunera.21@cse.mrt.ac.lk (S.S.); venura.21@cse.mrt.ac.lk (V.S.); dulanim@cse.mrt.ac.lk (D.M.); 2 School of Computer Science and Informatics, Cardiff University, Cardiff CF24 3AA, UK

**Keywords:** artificial intelligence, disaster management, multimodal RAG, sustainability, visual question answering

## Abstract

Wildfires are environmental hazards with severe ecological, social, and economic impacts. Wildfires devastate ecosystems, communities, and economies worldwide, with rising frequency and intensity driven by climate change, human activity, and environmental shifts. Analyzing wildfire insights such as detection, predictive patterns, and risk assessment enables proactive response and long-term prevention. However, most of the existing approaches have been focused on isolated processing of data, making it challenging to orchestrate cross-modal reasoning and transparency. This study proposed a novel orchestrator-based multi-agent system (MAS), with the aim of transforming multimodal environmental data into actionable intelligence for decision making. We designed a framework to utilize Large Multimodal Models (LMMs) augmented by structured prompt engineering and specialized Retrieval-Augmented Generation (RAG) pipelines to enable transparent and context-aware reasoning, providing a cutting-edge Visual Question Answering (VQA) system. It ingests diverse inputs like satellite imagery, sensor readings, weather data, and ground footage and then answers user queries. Validated by several public datasets, the system achieved a precision of 0.797 and an F1-score of 0.736. Thus, powered by Agentic AI, the proposed, human-centric solution for wildfire management, empowers firefighters, governments, and researchers to mitigate threats effectively.

## 1. Introduction

Wildfires have been a natural phenomenon over the years [1]. However, their frequency, intensity, and spatial extent have increased significantly in recent decades due to climate change and prolonged droughts [2]. Wildfires lead to natural disasters, such as landslides and flooding. causing environmental damage, public safety concerns, and economic damage [3]. In particular, large-scale land degradation and infrastructure loss cause significant financial losses. The exposure to delicate particulate matter from wildfire smoke can have immediate effects. Moreover, biomass combustion driven by wildfires contributes significantly to atmospheric carbon emissions [4,5]. As a result, this pollution reinforces climate change through feedback mechanisms [2]. These trends highlight the urgent need for more effective and adaptive wildfire monitoring and response strategies.

Traditional wildfire systems relied on physical models and rule-based systems [6,7,8]. As a result of their static nature, traditional wildfire systems faced limitations in handling complexity and performance. These systems typically rely on empirical and statistical analyses of fire weather indices, which have limited predictive power. With the limitations of these systems, traditional methods are rapidly being replaced by remote sensing technologies, Machine Learning (ML)-based fire detection algorithms, and advanced fire weather indices, resulting in significantly improved predictive accuracy [9,10].

With the transition to ML-based systems, Deep Learning (DL) plays a leading role. Studies have introduced approaches such as satellite image analysis with CNNs, integrating sensor systems with DL, and developing comprehensive systems that combine multimodal data [11,12,13,14]. Despite these advances, existing approaches exhibit several unresolved limitations. (1) Most systems process visual, meteorological, or textual data in isolation [15]. This leaves the problem of cross-modal integration largely unaddressed. (2) Many DL models function as opaque predictors, offering limited interpretability. This leads to a hindrance of trust and adoption in high-stakes decision-making contexts. (3) Prior MAS frameworks often assume stable communication infrastructures. However, the assumption does not hold in remote wildfire zones characterized by intermittent connectivity. (4) Current approaches rarely support human-in-the-loop reasoning. Nevertheless, it limits domain experts’ ability to interrogate, validate, or contextualize algorithmic recommendations.

While ML and DL have dominated artificial intelligence paradigms, emerging trends now leverage MAS integrated with LMM to enable collaborative, scalable, and context-aware decision-making in complex environments [16,17]. LMMs, alongside RAG, play a crucial role by augmenting reasoning capabilities through integration with external knowledge sources [18,19]. However, existing implementations typically operate under static and query-driven paradigms; thus, there remains a lack of mechanisms for dynamically orchestrating heterogeneous data streams in real time [6,20]. Consequently, their applicability to time-sensitive wildfire response scenarios remains limited. Notably, the proposed model must jointly interpret multimodal inputs, including aerial imagery, satellite data, and environmental sensor readings, under uncertain conditions.

The main objective of this study is to propose an orchestrated multi-agent framework with multimodal RAG pipelines to enable dynamic data integration, context-aware reasoning, and human-centered decision support for wildfire management. The proposed approach emphasizes coordinated reasoning across agents and modalities to support situational awareness in operational settings. Accordingly, this study addresses the following research questions:

(RQ1) How can an orchestrator-based MAS dynamically integrate heterogeneous data sources (such as UAV imagery, satellite observations, and tabular environmental data) into a unified reasoning framework for wildfire response?
(RQ2) What advantages does a multimodal MAS integrated with RAG pipelines offer over traditional wildfire prediction and management approaches?

Consequently, this study makes the following key contributions to the domain of wildfire management.

### Statement of Novelty and Contributions

While recent works like WildfireGPT [15] and SmokeyNet [14] have applied AI to wildfire management, our work introduces three distinct architectural innovations that differentiate it from existing RAG-based and multi-agent paradigms:

1. Architectural Determinism via Decay-Weighted Routing: Unlike standard autonomous agents that rely on open-ended loops (which are prone to “getting stuck”), we introduce a novel orchestration policy $\pi (q,C_{t})$ governed by a decay factor. This mathematically enforces task convergence, a critical requirement for safety-critical response systems.
2. Lossless Multimodal RAG: We address the information loss inherent in standard “caption-based” retrieval systems. By retrieving and processing raw visual artifacts (Base64) rather than text descriptions, our pipeline preserves the forensic granularity required to distinguish between smoke plumes and cloud cover.
3. Formalized State Engineering: We replace standard natural language prompting with a rigorous “Context Tuple” framework (*p*). This formalization constrains the stochastic nature of Large Multimodal Models (LMMs), ensuring that agent behavior is reproducible and auditable, a feature largely absent in generic generative AI frameworks.

By advancing multimodal, context-aware and human-centered decision-support mechanisms, this work contributes to the development of more resilient wildfire response systems. It aligns with the united nations sustainable development goals on sustainable cities (SDG11), climate action (SDG 13), and life on land (SDG 15) by advancing technologies for disaster resilience and ecosystem preservation [21].

## 2. Related Work

Early research in wildfire management primarily relied on physics-based and rule-based models to simulate fire behavior and estimate fire danger. These approaches typically model wildfire spread as a wave-propagation process, governed by meteorological variables such as wind speed, temperature, and fuel characteristics [22]. For instance, Tavakol et al. [6] demonstrated the use of UAV swarms that employ simple rule-based heuristics for autonomous wildfire suppression. From another point of view, several studies have focused on handling environmental uncertainty and improving response times through fuzzy logic and edge computing. Duarte et al. [7] developed a Fuzzy Inference System (FIS) to map forest fire susceptibility in the Amazon region, utilizing fuzzy rules to effectively model the ambiguity inherent in meteorological and land-use data. Similarly, Toledo-Castro et al. [8] proposed a dynamic fuzzy logic controller embedded within wireless sensor networks, enabling distributed nodes to autonomously validate fire outbreaks and reduce false alarms without relying on a central server. While such models offer valuable theoretical insights into wildfire dynamics, they exhibit certain limitations such as (1) an inability to consistently model fire behaviour across different spatial and temporal scales, (2) high data requirements and computational expense, and (3) challenges in validating model predictions against real-world fire phenomena. In real-world wildfire scenarios, where data are incomplete, delayed, or noisy, these requirements are rarely met. Consequently, physics-based models offer limited support for real-time decision-making during rapidly evolving fire events [23,24].

To overcome the limitations of traditional modeling approaches, recent studies have increasingly adopted data-driven methods based on DL [11,12,25,26]. A prominent example of this is “SmokeyNet” [14], a DL-based model that integrates Convolutional Neural Networks (CNNs), Long Short-Term Memory Networks (LSTMs), and Vision Transformers (ViT) to identify smoke patterns from multiple sources, including the HP- WREN camera network in the USA. Although such studies have shown high performance in specific tasks like smoke detection, they lack the cognitive capabilities to forensically identify the significance. Another study, WildfireGPT [15], primarily handles textual queries with climate projections and knowledge retrieval, lacking dynamic orchestration to fuse multimodal inputs like UAV imagery with sensor data in evolving scenarios, resulting in delayed contextual insights during time-sensitive events.

Another important consideration is the explainability in environmental data screening with remote sensing, which is crucial for building trust in AI-driven decisions that impact disaster management, climate monitoring, and ecosystem preservation [27,28,29]. The study by Klotz et al. [29] addressed the effectiveness of explainable AI (XAI) methods and evaluation metrics for remote sensing image scene classification. The study provided methodological insights and experimental validation, offering guidelines for selecting appropriate XAI methods, metrics, and hyperparameters tailored to remote sensing contexts. Similarly, Ahangama et al. [28] proposed XAI framework for precipitation nowcasting using spatio-temporal and multivariate image data to predict short-term rainfall patterns with pixel-level accuracy. The generated attribution maps have revealed the model’s focus on critical features like echo intensity and motion patterns across image sequences, enhancing trust and interpretability for meteorological decision-making in disaster-prone regions.

Recently, the emergence of Agentic AI and Generative AI has introduced new paradigms for autonomous and interactive disaster management [30,31,32]. MAS have been widely explored for decentralized coordination. For example, Zadeh et al. [20] integrated Deep Reinforcement Learning (DRL) into a high-level MAS to optimize fire tracking, while Mawanza [18] proposed a heterogeneous MAS combining UAVs and ground robots for comprehensive monitoring. Additionally, Kouzehgar et al. [19] explored Multi-Agent Reinforcement Learning (MARL) for swarm-based ocean monitoring, a technique with high transferability to wildfire detection scenarios.

Moreover, studies have addressed edge-based solutions as well. For instance, addressing the connectivity and bandwidth limitations of remote monitoring, Kalatzis et al. [33], introduced a hierarchical edge computing framework for UAVs. By processing visual and infrared data directly on ‘Edge’ nodes rather than transmitting raw streams to a cloud server, their system significantly reduces the latency typically associated with centralized processing.

While frameworks like WildfireGPT [15] utilize LLMs for textual queries, they rely on static retrieval workflows that lack dynamic cross-modal synthesis. Furthermore, standard multi-agent orchestration paradigms often assume stable connectivity and prioritize open-ended exploration. In contrast, our architecture introduces a ‘Strategic Real-Time’ constraint. We deviate from standard agent frameworks by implementing strict logical guardrails and a weighted intent-match formula that prioritizes safety and interpretability over the creative autonomy typically found in Generative AI agents.

To provide an unambiguous distinction between the proposed system and existing state-of-the-art frameworks (such as WildfireGPT and standard ReAct agents), we present a comparative analysis in Table 1. This comparison highlights the specific architectural deviations, such as the shift from recursive looping to decay-weighted routing and from caption-based retrieval to lossless artifact injection, that are necessary to ensure the reproducibility and precision required for wildfire insights.

Table 2 and Table 3 provide an overview of existing studies that have utilized different approaches over the years, in related domains.

As shown in Table 3, several limitations persist in existing wildfire management support studies. The primary gap is the lack of cross-modal integration, as most models handle visual or meteorological data independently. Another issue is that existing solutions provide limited contextual decision support, where DL models predict fire spread, but the interpretability or situational guidance for responders is not sufficient. Additionally, scalability and communication bottlenecks are challenges in current solutions. Several MAS architectures assume perfect connectivity, which is impractical in remote fire zones with unstable networks. Furthermore, there is minimal human-in-the-loop reasoning, failing to provide firefighters and planners with the transparent, conversational systems needed to justify and explain recommendations. Accordingly, the comparison of wildfire screening approaches reveal that numerous studies have employed traditional remote sensing techniques alongside ML and DL; however, the integration of MAS with RAG and LMMs remains underexplored and represents an emerging research frontier. In order to overcome these gaps, the proposed study enables agents to collaboratively interpret multimodal data, reasoning through natural language, and deliver context-aware decision support for wildfire management.

## 3. Methodology

### 3.1. Process Overview

**Figure 1:**
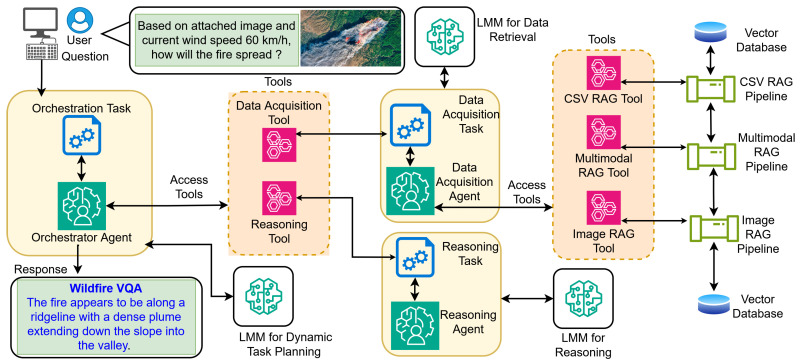
High-level architectural diagram of the proposed solution.

The proposed framework employs a hierarchical MAS that decouples task orchestration from execution, as depicted in the system architecture shown in Figure 1.

A central orchestrator agent dynamically manages the full lifecycle of a user query, from ingestion to response generation. This design overcomes the constraints of linear pipelines by supporting conditional branching and adaptive tool selection. The process initiates when a multimodal query that combining natural language text with images such as satellite or UAV imagery is submitted via the VQA interface. The input is routed directly to the orchestrator agent, which acts as the core decision-making hub. Unlike rigid static workflows, the orchestrator leverages an LMM to parse the query’s semantic intent. It then selects the optimal execution path among subordinate agents, distinguishing simple retrieval tasks from those requiring multi-step reasoning. When external environmental context is required, the orchestrator invokes the data acquisition agent via the data acquisition tool. This specialized agent serves as the interface to the underlying RAG infrastructure. In order to handle heterogeneous data formats, the data acquisition agent is equipped with three distinct retrieval mechanisms:

- CSV data retrieval pipeline: retrieves structured meteorological and historical wildfire data from a vector database using high-dimensional text embeddings.
- Image data retrieval pipeline: retrieves visual analogs from satellite or aerial imagery databases, utilizing visual embeddings to identify patterns similar to the input image.
- Multimodal RAG pipeline: synchronizes the retrieval of both text and image data when the query necessitates a combined environmental view.

This modular separation isolates raw data processing from higher-level reasoning logic. Once relevant data artifacts are retrieved and aggregated, control returns to the orchestrator, which activates the reasoning agent via the reasoning tool. The reasoning agent processes the aggregated context, comprising retrieved metadata and the original query, through an LMM to generate the final reasoned output. The orchestrator then formats this output and delivers it to the user interface, completing the interaction loop.

### 3.2. Materials and Datasets

This study leverages wildfire and environmental datasets, as shown in Table 4. The datasets are from public sources across multiple geographical regions, including Algeria [34], the United States [35,36], and Canada. These datasets were selected based on data availability and the significant ecological impact of wildfires in these areas.

To ensure a rigorous and fair comparison between different LMM backbones, a strict hold-out evaluation procedure has been employed. The primary datasets FLAME 3 and Algerian Forest Fires have been split into two mutually exclusive subsets. The first subset has been used only to populate the RAG vector indices ($Index_{tab}$, $Index_{img}$) and select the few-shot exemplars (ϵ) used in context engineering. This subset represents the historical context that is accessible to the agents during their reasoning process. The second subset, referred to as the Evaluation Subset or Test Set, represents a reserved subset of instances used exclusively for final testing and benchmarking. This subset has been withheld from the vector database and used to simulate unseen wildfire scenarios used in testing.

The splitting of the datasets has been performed in a non-dynamic manner. A single test set was also created before any experimentation took place, and all models were evaluated using this same test set. This means that models such as GPT-4o, GPT-5-Nano, etc. have all been tested using the same unseen instances. This is in contrast to random re-sampling a test set for each different experimental run. This procedure ensures that all models have been tested under exactly the same conditions, so any variance in performance reported in Section 4 is solely due to the capabilities of each LMM backbone.

### 3.3. Data Preprocessing and Retrieving

The preprocessing pipeline addresses heterogeneous data sources, while ensuring quality, consistency, and compatibility with downstream retrieval and generation components. It is customized to the specific characteristics of the employed datasets.

#### 3.3.1. Preprocessing and Retrieval Algorithms

Below are the algorithmic procedures for data preprocessing (Algorithm 1) and multimodal retrieval with generation (Algorithm 2). For a complete list of the mathematical notations and definitions used in these algorithms, please refer to Appendix B.
**Algorithm 1** Multimodal RAG pipeline: preprocessing phase**Require:** Tabular dataset $D_{tab}$, Image dataset $D_{img}$**Ensure:** Populated indices ${\mathrm{Index}}_{tab}$, ${\mathrm{Index}}_{img}$    **Hyperparameters:**1:$\tau_{miss}\leftarrow 0.40$         ▹ Threshold for dropping rows with missing values2:$\theta_{iqr}\leftarrow 1.5$            ▹ Multiplier for IQR-based outlier detection3:**procedure** PreprocessTabular($D_{tab}$)4:      **for** each row $r_{i}\in D_{tab}$ **do**5:          **if** $\mathrm{MissingRatio}\left(r_{i}\right)>\tau_{miss}$ **then**6:              $D_{tab}\leftarrow D_{tab}-\left\{r_{i}\right\}$     ▹ Remove row if too much data is missing7:          **else**8:              $r_{i}\leftarrow \mathrm{Impute}\left(r_{i},\mathrm{method}=\left\{\mathrm{mean},\;\mathrm{mode}\right\}\right)$9:              $r_{i}\leftarrow \mathrm{RemoveOutliers}\left(r_{i},\theta_{iqr}\right)$10:             $r_{i}\leftarrow \mathrm{Normalize}\left(r_{i},Z-\mathrm{score}\right)$11:             $t_{i}\leftarrow \mathrm{SerializeToText}\left(r_{i}\right)$        ▹ Convert row data to text string12:             $v_{i}\leftarrow {\mathrm{Model}}_{BAAI}\left(t_{i}\right)$            ▹ Generate 768-dim embedding13:           ${\mathrm{Index}}_{tab}.\mathrm{add}\left(v_{i},\mathrm{metadata}\left(r_{i}\right)\right)$14:        **end if**15:    **end for**16:**end procedure**17:**procedure** PreprocessImages($D_{img}$)18:    **for** each image $I_{j}\in D_{img}$ **do**19:        **if** $\neg \mathrm{IntegrityCheck}(I_{j})$ **or** $\mathrm{IsDuplicate}(I_{j})$ **then**20:           $D_{img}\leftarrow D_{img}-\left\{I_{j}\right\}$         ▹ Discard corrupt or duplicate images21:        **else**22:           $I_{j}\leftarrow \mathrm{Resize}\left(I_{j}\right)$              ▹ Normalize image resolution23:           $u_{j}\leftarrow {\mathrm{Model}}_{CLIP}\left(I_{j}\right)$            ▹ Generate visual embedding24:           ${\mathrm{Index}}_{img}.\mathrm{add}\left(u_{j},\mathrm{metadata}\left(I_{j}\right)\right)$25:        **end if**26:    **end for**27:**end procedure**

**Algorithm 2** Multimodal RAG pipeline: retrieval and generation phase**Require:** User query *q*, Populated indices ${\mathrm{Index}}_{tab}$, ${\mathrm{Index}}_{img}$**Ensure:** Generated answer *A*        **Hyperparameters:**
1:k←Top-kretrievalcount       ▹ Number of items to retrieve per search
2:**procedure** MultimodalGeneration(*q*)3:    $q_{vec}\leftarrow {\mathrm{Model}}_{BAAI}\left(q\right)$         ▹ Embed user query for text search4:    $C_{tab}\leftarrow {\mathrm{Index}}_{tab}.\mathrm{SearchKNN}\left(q_{vec},k\right)$       ▹ Retrieve tabular context5:    $q_{clip}\leftarrow {\mathrm{Model}}_{CLIP}.\mathrm{EncodeText}\left(q\right)$     ▹ Embed query for image search6:    $C_{img}\leftarrow {\mathrm{Index}}_{img}.\mathrm{SearchKNN}\left(q_{clip},k\right)$       ▹ Retrieve visual context7:    $C_{aug}\leftarrow \mathrm{PromptAugment}\left(q,C_{tab},C_{img}\right)$      ▹ Combine retrieved contexts8:    $A\leftarrow {\mathrm{Model}}_{Gen}\left(C_{aug}\right)$               ▹ Generate final reasoning9:    **return** *A*10: **end procedure**


**Figure 2:**
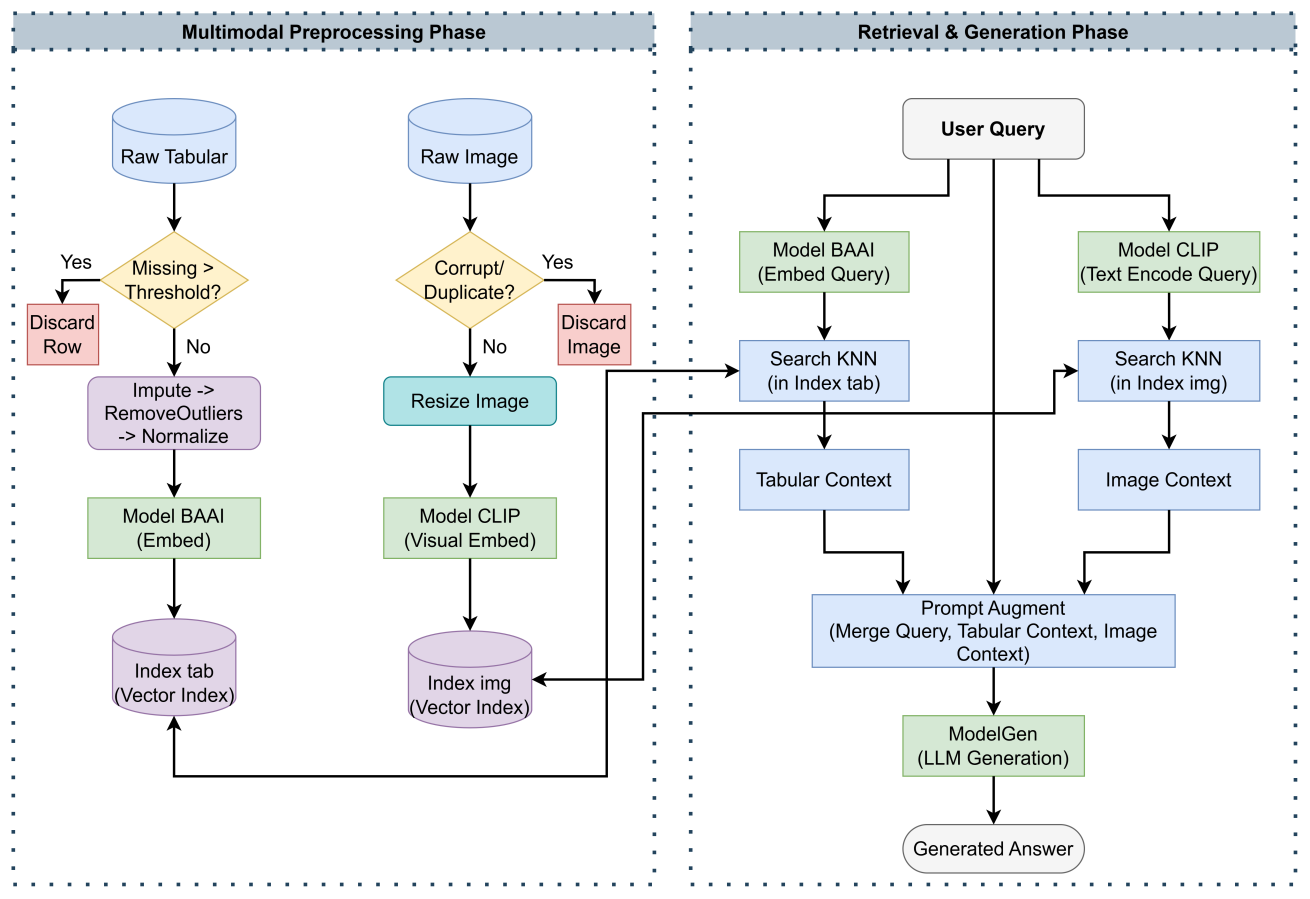
Architectural overview of the Multimodal RAG Framework.

Figure 2 provides a high-level architectural view of the multimodal RAG pipeline, highlighting the interaction between preprocessing, vector indexing, multimodal retrieval, and LLM-based generation.

Furthermore, the retrieval depth parameter was set to k=20 in order to maximize the level of retrieval in terms of recall within the context-retrieval phase. For wildfire management, it is important to maximize diversity in terms of historical analogs, especially in terms of retrieval numbers, in order to capture specific edge cases that could otherwise be lost with a lower number of retrieval counts. This design choice operates on the premise that the reasoning agent’s LMM (e.g., GPT-5-Nano) possesses sufficient attention mechanisms to effectively filter contextual noise from a larger pool of retrieved artifacts. As such, k=20 was determined to be optimal in terms of retrieval in order to provide a wide enough scope of meteorological and visual scenarios within the context window of the prompt.

#### 3.3.2. Pipeline Execution Flow

Algorithms 1 and 2 outline the process within pre-processing, retrieval and generation phases. The process starts with the processing of the raw tabular dataset $D_{tab}$, which contains meteorological and fire data, and the raw image dataset $D_{img}$, comprising satellite or aerial imagery. The procedure iterates through the tabular dataset, removing rows $r_{i}$ with a missing value ratio exceeding the threshold $\tau_{miss}$ of 0.40, followed by statistical imputation and the removal of outliers using the interquartile range multiplier $\theta_{iqr}$, which is 1.5. Subsequently, each valid row is serialized into a textual format $t_{i}$ and transformed into a vector embedding $v_{i}$ using the transformer model $Model_{BAAI}$ (BAAI/bge-base-en-v1.5), which is then stored in the tabular vector index $Index_{tab}$. In parallel, the system analyzes the image $I_{j}$ from the dataset $D_{img}$, evaluating integrity checks that remove redundant and corrupted images, followed by resizing and the computation of the image embedding $u_{j}$ by the $Model_{CLIP}$ (OpenAI CLIP ViT-B/32) transformer model, for storage in the image index $Index_{img}$.

To address the challenge of aligning different embedding spaces (BAAI/bge-base-en-v1.5 embedding space and CLIP embedding space) without training a computationally expensive bridge encoder or projection layer, the system adopts a contextual prompt augmentation strategy instead of joint retrieval in a shared vector space. When a user provides a natural language query *q*, the textual component is converted into embeddings $q_{vec}$ using $Model_{BAAI}$ to query the tabular index $Index_{tab}$. In order to increase the accuracy of these primary retrieval results, a re-ranking process is carried out using the Cohere 5.11.1 Re-rank model to produce the final tabular context $C_{tab}$. Meanwhile, the querying process is carried out in $Model_{CLIP}$ to produce $q_{clip}$ to query visually similar elements $C_{img}$ from the image index $Index_{img}$. Due to the involvement of different modalities in different vector spaces, parallel streams are utilized in retrieval.

The alignment and synthesis of these heterogeneous data points take place during the augmentation step. Specifically, this process involves the deterministic construction of a composite input sequence, where the user query *q*, the serialized tabular records $C_{tab}$, and the retrieved visual artifacts $C_{img}$ are concatenated within a structured prompt template. A distinguishing feature of our multimodal RAG pipeline, compared to existing cross-modal retrieval frameworks, is the preservation of visual fidelity. Rather than generating intermediate textual captions, which acts as a lossy compression step, the system retrieves raw image artifacts in Base64 format. These are interleaved with tabular context in the augmented prompt $C_{aug}$, allowing the LMM to utilize its native cross-attention mechanisms on the original pixel data, yielding higher precision in identifying specific burn patterns.

Consequently, the proposed framework operates strictly at the representation level of information fusion. Unlike decision-level systems that aggregate independent probability scores from isolated unimodal models, our architecture interleaves raw visual encodings and serialized text within the LMM’s shared context window ($C_{aug}$). This enables the model to perform deep semantic fusion via cross-modal attention layers during the inference phase, mapping heterogeneous inputs into a unified latent representation space prior to generating the final reasoning output.

### 3.4. Structured Prompt Engineering and Context Formalization

In order to mitigate the stochastic nature of LMMs and ensure deterministic task execution, the proposed solution employs a structured prompt engineering framework. Rather than solely relying on natural language instructions, it formalizes the prompt space as a specialized instantiation of context engineering [38,39].

A pre-defined prompt p∈P is structured as a tuple p=(ρ,γ,τ,ε,ϕ), where ρ∈R denotes the role specification, defining the agent’s identity and strictly bounded domain expertise to prevent out-of-scope task execution; γ∈G represents the goal state of the agent, defining the objective function the agent must optimize; τ∈T corresponds to the task vector, which is a sequence of executable instructions required to achieve γ; ε∈E represents reference cases, providing ground-truth input–output pairs to guide the model’s reasoning trajectory. These cases are strictly curated from the training and validation subsets of the primary datasets (FLAME 3 and Algerian Forest Fires) to serve as few-shot exemplars. Specifically, we employ a static set of three exemplars for each agent. Unlike dynamic exemplar retrieval, which can result in different styles of reasoning depending on the retrieved exemplars, a static set guarantees deterministic behavioral convergence. In this way, the agent avoids prompt drift and ensures that it strictly adheres to the safety protocols with each cycle of execution. Importantly, to prevent data leakage and ensure the validity of the evaluation, there is no overlap between these reference cases and the independent test set used to generate the quantitative performance metrics presented in Section 4.4; ϕ∈C specifies operational constraints, including negative constraints (what not to do) and strict output formatting rules (e.g., JSON schemas) to ensure inter-agent communication compatibility. These context parameter values are provided for each agent before executing. While executing, agents can work along with these given context parameters.

This formalism is applied distinctively across the agentic workflow. For the orchestrator agent, the constraint set ϕ enforces a strict routing syntax to enable dynamic task decomposition without hallucinating non-existent tools. Similarly, for the reasoning agent, the task vector τ maintains a Chain-of-Thought (CoT) process to ensure that all outputs are explainable and grounded in the retrieved artifacts [40]. To resolve potential conflicts between heterogeneous data sources (e.g., a meteorological record suggesting low risk vs. visual evidence of a plume), the CoT prompt implements a ‘Hierarchical Evidence Weighting’ protocol. This instructs the reasoning agent to assign higher validity to direct observational evidence (raw visual artifacts from the Multimodal RAG pipeline) over static or predicted metadata (from the CSV pipeline) when identifying active hazards.

### 3.5. Proposed Multi-Agent Architecture

This study experimented with the following two architectural paradigms when implementing the proposed solutions.

1. Task-Based Approach
2. Orchestrator-Based Approach

Generally, the proposed MAS is formally defined as a tuple M=(A,T,L,R), denoting Agent, Task, Tool and Rule, respectively, which are described as follows.

- Agent A={OrchestratorAgent,DataAcquisitionAgent,ReasoningAgent} denotes the set of agents in the system. An Agent is an intelligent and automated unit powered by an LMM that performs specific tasks. Apart from the LMM, an agent must be provided with a role, a goal describing its instructions, a set of tasks to achieve the aforementioned goal, and a set of tools it can use to perform its assigned tasks. Agents can establish communication with other agents while maintaining their own memory of interactions. An output of an agent depends on the LMM it is supplied with. Therefore, the most suitable LMM for an agent may vary, and it largely depends on the tasks assigned to the agent. The proposed solution has three key agents that support its functionality.
  – Data acquisition agents gather data for processing. This agent is supplied with data retrieval tools, including the CSV data retrieval tool, the Image data retrieval tool, and the multimodal RAG tool, and invoke them when necessary.
  – The reasoning agent processes complex spatial–temporal patterns in wildfire behavior, analyzing the wildfire cases that have previously happened. Generally, this agent is invoked after the data acquisition agent is invoked, because the data acquisition agent provides the data that is retrieved from its multiple RAG pipelines. Then the reasoning agent provides the reasoning about the current situation with the data that it gets from the Data Acquisition agent.
  – Orchestrator agent coordinates tasks and their execution within this MAS. As the central coordinator of the proposed framework, it enables full functionality by connecting other intelligent agents as tools. As shown in Figure 1, the data acquisition agent serves as the data acquisition tool, and the reasoning agent serves as the reasoning tool. The orchestrator agent facilitates communication between these sub-agents, allowing seamless task delegation. Its plan-and-execute nature provides adaptability to dynamic environments, making it ideal for domains such as wildfire management.
  – Structurally, the system adopts a hub-and-spoke (Star) topology, enforcing strict isolation between subordinate agents. The data acquisition agent and reasoning agent operate in distinct environments and are invoked via independent API calls. Consequently, they possess no shared memory or lateral communication channels; the state of the orchestrator is opaque to them. This design ensures that the orchestrator agent acts as the sole source of truth for conversation history and global context, preventing information leakage and ensuring that all inter-agent data flow is explicitly filtered and routed through the central policy function.
- Task $T=\{t_{1},t_{2},\ldots ,t_{n}\}$ represents the set of tasks, which are dynamically planned and executed by the Orchestrator Agent.
- Tool L={CSVdataretrievaltool,Imagedataretrievaltool,MultimodalRAGtool} represents the set of functions available within the system. Each tool is a specialized skill or capability that agents can invoke to execute specific actions. Beyond pre-built tools, custom tools can be developed and assigned to agents to extend their functionality. As shown in Figure 1, these tools are primarily utilized by the data acquisition agent and custom-built as follows.
  – The CSV data-retrieval tool is an application interface that utilizes a CSV-based RAG Pipeline to retrieve raw metadata from indexed meteorological CSV data.
  – The image data retrieval tool is an application interface that utilizes an Image Data Retrieval pipeline. It accepts both text and image inputs as queries and retrieves relevant images in base-64 format along with their metadata.
  – The multimodal RAG tool utilizes the above retrieval pipelines in parallel. This ensures that the Reasoning Agent receives context that is both semantically and visually grounded.
- Rule *R* represents the set of interaction rules governing agent coordination. These rules are specified independently of the agent prompts.

#### 3.5.1. Task-Based Architecture

**Figure 3:**
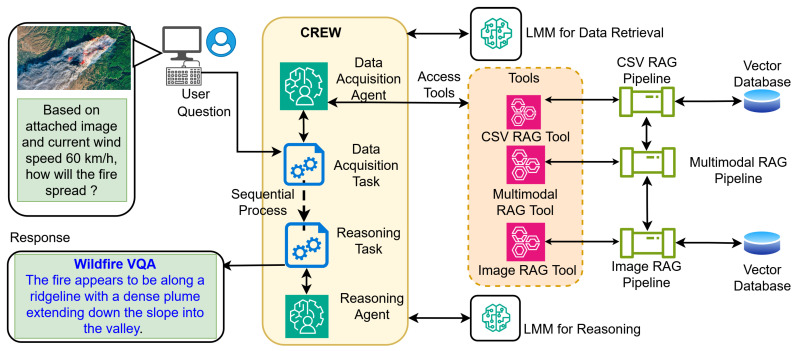
System Architecture Overview Diagram of the Task-Based Approach.

As shown in Figure 3, the task-based approach is modeled as a linear composite function. Let $A_{data}$ and $A_{reason}$ denote the data acquisition agent and the reasoning agent, respectively. Then, the system response *S* to a posed user query *q* can be stated as in (1).

(1) $$R=A_{\mathrm{reason}}\left(A_{\mathrm{data}}\left(q\right)\right)$$

Figure 3 reflects the “sequential process”, where the output of the data acquisition task is passed as the input for the reasoning task. Here, the probability of a successful response $P(R_{success})$ is strictly dependent on the joint probability of success for both sequential steps, as given in (2).

(2) $$P\left(R_{success}\right)=P\left(success|A_{data}\right)\cdot P\left(success|A_{reason}\right)$$

Therefore, this task-based approach has the following limitations.

1. Error propagation: since the coupling is linear (R=f(g(x))), any hallucination in the initial retrieval stage is propagated to the reasoning stage, which can lead to erroneous outcomes.
2. Computational inefficiency: as per (1), this approach forces the execution of $A_{data}$ for every query. This results in an inevitable computational cost $Cost(A_{data})$, even when the retrieved data might not be of use (e.g., for general knowledge queries).

Accordingly, we propose an orchestrator-based approach to address the limitations of the task-based approach.

#### 3.5.2. Orchestrator-Based Architecture

The orchestrator-based approach, as shown in Figure 1, redefines the system as a dynamic decision process. Unlike the fixed sequence in Figure 3, the orchestration agent node acts as a central policy function π that maps the current query *q* and current context at a given time *t* ($C_{t}$) to an optimal target agent. The routing decision is modeled as an optimization problem where the orchestrator selects an agent $a_{target}$ to maximize a relevance score *S*, such that $\pi \left(q,C_{t}\right)\to \left\{a_{target},T_{tools}\right\}$.

Let, IntentMatch quantifies the semantic similarity between the user’s intent and the agent’s functional role; DataNeed represents the necessity of specific modalities (e.g., a thermal image query explicitly weights the data acquisition agent); and α and β are adaptive weighting coefficients derived from the system prompt. Then, the selection of $a_{target}$, represented by the “Access Tools” arrows in Figure 1, is determined by (3).

(3) $$a_{target}=\underset{a\in A}{\mathrm{argmax}}\left(\alpha \cdot \mathrm{IntentMatch}\left(q,{\mathrm{Role}}_{a}\right)+\beta \cdot \mathrm{DataNeed}\left(q,{\mathrm{Capability}}_{a}\right)\right)$$

The core novelty of our orchestration lies in the dynamic policy optimization that balances ‘Intent Match’ (α) against ‘Data Need’ (β). Unlike standard semantic routers that use a simple cosine similarity threshold, our Orchestrator dynamically instantiates these weighting coefficients based on query complexity. Crucially, we introduce a decay factor $\lambda_{decay}$ (Algorithm 3, Line 20). This algorithmic modification prevents the infinite retrieval loops often observed in recursive agent frameworks, ensuring the system converges toward a reasoning state, a necessary architectural adaptation for emergency response systems.

The architectural decoupling of the data acquisition agent with the reasoning agent creates an artificial latency bottleneck that prevents the unverified discharge of assessment-level information. The application of the ‘decay-weighted’ routing policy $\pi (q,C_{t})$ ensures the completion of the data retrieval cycle, which includes the cross-referencing of CSV artifacts, before the reasoning agent begins the process of creating an assessment-level response. This increases the time-to-insight for the user by approximately 176 s but acts as a logical gatekeeper to the unverified discharge of potentially hallucinated advice.

The routing logic relies on the dynamic maximization of the relevance score $S_{a}$. As defined in Algorithm 3, Line 3, the weighting coefficients α (Intent Match importance) and β (Data Need importance) are not hard-coded constants but are dynamically instantiated by the Orchestrator’s LMM upon initial query analysis. The function $\pi (Q,C_{t})$ represents the policy that maps the current query *Q* and context $C_{t}$ to the optimal agent.
**Algorithm 3** Orchestrator dynamic policy optimization**Require:** User query *Q*, Set of available agents A, Initial context $C_{0}$**Ensure:** Final system response *R*  **Parameters:** Decay rate $\lambda_{decay}$, Threshold τ1: $C_{t}\leftarrow C_{0}$2: R←NULL3: α,β←InitializeWeights(Q)     ▹ Derived via LLM analysis of query intent4: **while** *R* is NULL **do**5:     $S_{max}\leftarrow -\infty$6:     $a_{target}\leftarrow \mathrm{NULL}$7:     **for** each agent a∈A **do**           ▹ The policy function $\pi (Q,C_{t})$8:         $I_{match}\leftarrow \mathrm{ComputeIntentMatch}\left(Q,{\mathrm{Role}}_{a}\right)$9:         $D_{need}\leftarrow \mathrm{ComputeDataNeed}\left(Q,C_{t},{\mathrm{Capability}}_{a}\right)$10:        $S_{a}\leftarrow \left(\alpha \cdot I_{match}\right)+\left(\beta \cdot D_{need}\right)$        ▹ Calculate optimization score11:        **if** $S_{a}>S_{max}$ **then**12:             $S_{max}\leftarrow S_{a}$13:                $a_{target}\leftarrow a$14:         **end if**15:    **end for**16:     **if** $a_{target}==\mathrm{Data}\;\mathrm{acquisition}\;\mathrm{agent}$ **then**17:         $T\leftarrow \mathrm{SelectTools}(a_{target},Q)$18:         Data←ExecuteTools(T)19:         $C_{t}\leftarrow C_{t}\cup Data$         ▹ Update context with retrieved artifacts20:         $\beta \leftarrow \beta \cdot \lambda_{decay}$           ▹ Decay data weight to force convergence21:    **else if** $a_{target}==\mathrm{Reasoning}\;\mathrm{agent}$ **then**22:         $R\leftarrow \mathrm{GenerateResponse}(a_{target},Q,C_{t})$23:    **else**24:         R←DirectResponse(Orchestrator,Q)         ▹ Fallback for queries25:    **end if**26: **end while**27: **return** *R*

The InitializeWeights(Q) function represents a zero-shot prompt analysis, where the LMM assigns higher values to β the query *Q* contains explicit requests for external data (e.g., “Show me current wind speeds”) and higher values to α for logic-heavy queries.

To mitigate the inherent stochasticity of LMMs and ensure consistent routing behavior, strict deterministic guardrails were implemented for the orchestrator agent. First, the model’s temperature parameter was set to 0.0, effectively forcing the model to greedily sample the most probable token, thereby minimizing variance in weight generation (α,β). Second, the output space of the orchestrator was constrained using structured decoding and logit bias enforcement. That is the, bias occurs due to the unnormalized output score from the neural network’s final layer before applying activation functions like softmax to convert the output into probabilities. This restricts the generated weights to a discrete numerical range and enforces a strict JSON schema for tool selection, preventing format hallucinations. These mechanisms ensure that the policy function $\pi (q,C_{t})$ remains mathematically stable across independent execution runs for identical queries.

In addition to the pseudocode provided by Algorithm 3, Table 5 shows the actual decision boundaries used by multi-agent system. It establishes the unique query conditions with the major weighting factors (α,β) and the corresponding execution flow, showing the enforcement of the Strategic Real-Time constraints.

Given that agents operate as independent API endpoints, the orchestrator agent implements a supervisor–worker validation protocol to handle inconsistent or low quality outputs. Since subordinate agents are stateless, the orchestrator can dynamically re-invoke them with refined parameters without resetting the entire system state. If an agent yields conflicting information, such as the data acquisition agent returning null meteorological data or if an agent exhibits poor reasoning (hallucinated formats), the orchestrator triggers a recursive correction loop. This mechanism allows the system to resolve conflicts by prioritizing grounded evidence over incomplete retrieval results, ensuring that temporary failures in one environment do not propagate as final system errors.

#### 3.5.3. Comparison of Architecture

Table 6 shows a comparison between the two approaches, where the orchestrator-based method observes better maintainability, informing the decision to adopt it for scalable, adaptive wildfire management.

#### 3.5.4. Application Implementation

**Figure 4:**
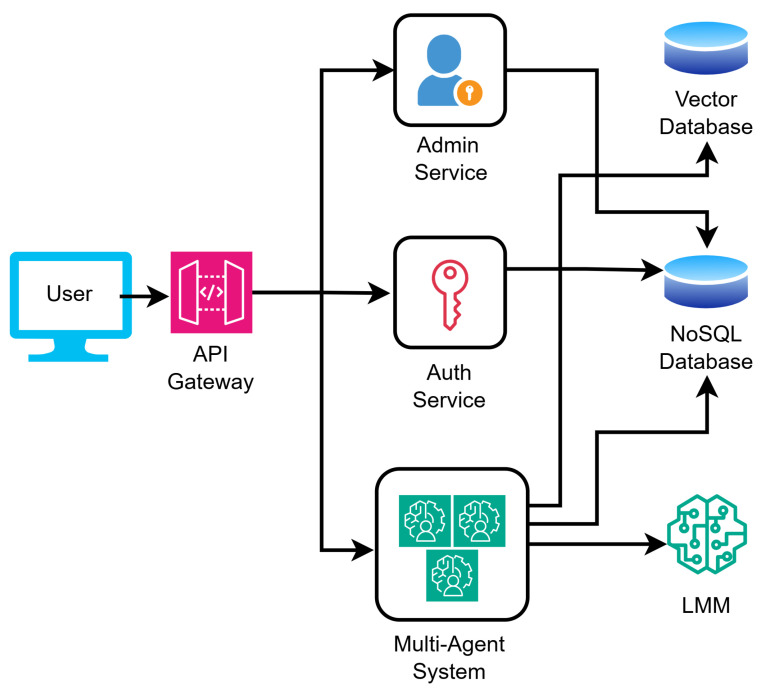
High-level Architectural Diagram of the application.

Figure 4 illustrates the high-level application architecture, where an API Gateway routes user requests to the core Multi-Agent System for wildfire reasoning, alongside authentication and admin services. This backend integrates Large Multimodal Models (LMMs) for intelligence, utilizing Vector and NoSQL databases (MongoDB 7.0.28) for efficient multimodal data retrieval and secure session management.

## 4. Results

### 4.1. Applied Task Formulation

In order to properly assess and evaluate the effectiveness of the MAS, a multimodal wildfire reasoning and response task was formulated. The specific subtasks comprising this formulation (hazard identification, spread prediction, and response planning) are detailed in Table A1 in Appendix A. This task was designed to simulate the decision-making process of an incident commander under dynamic operational conditions. Adhering to the protocol defined in Section 3.2, a strict hold-out evaluation approach was maintained. The data is split in a manner such that none of the data used in creating the vector embeddings or few-shot prompts will ever enter the test set. To ensure a proper comparison between different LMM backbones, a common test set is used in all evaluations without dynamic re-sampling, ensuring that the reported metrics reflect true architectural differences rather than data variance.

The system’s objective extends beyond binary hazard detection (“Fire” vs. “No Fire”). For each test instance, the system is engaged in a process of multiple-step reasoning that entails the identification of the presence of live fire threats through a cross-checking of visual artifacts with meteorological information, prediction of the direction and rate of spread of fire based on wind direction information and terrain data, and the development of particularized mitigatory action plans based on synthesized information. While the quantitative measures of Accuracy, Precision, Recall, and F1-Score presented in this section of the paper primarily measure the system’s grounding and detection capacity, the quality of the system’s reasoning is measured through a qualitative assessment of the system’s generated responses.

With respect to the statistical analysis of variance, this research focused on deterministic behavior to ensure the level of reliability required for disaster response. As highlighted in the orchestration policy, the LMMs employed a temperature setting of 0.0 to ensure greedy sampling, thus eliminating the level of stochastic behavior within the generated outputs of the agents. As a result, the outputs are similar even if the models are executed several times on the same hold-out test set. Thus, the level of variance is negligible, and the evaluation strategy was focused on ensuring a strict architectural comparison, eliminating the impact of the random seed variations that are normally associated with generative models.

The reliability of the generated insights is validated through the architectural enforcement of the orchestrator’s recursive validation loop, not through the statistical averaging of stochastic repetitions. Unlike standard generative evaluations that quantify reliability via confidence intervals, this system utilizes an iterative self-correction mechanism to ensure the output converges to a stable, logic-compliant state. This approach effectively replaces the need for traditional confidence analysis based on repetition with architectural determinism. The system is designed to produce a optimal response for any given input. By having this operational consistency, the provided architecture guarantees that identical environmental variables trigger a uniform response.

### 4.2. Evaluation Metrics

We assessed the proposed solution utilizing a suite of metrics tailored to wildfire prediction and detection tasks. Core classification metrics, including accuracy, precision, recall, and F1-score, assess model reliability, guiding agent selection (e.g., GPT-5-Nano) to prioritize high precision in scenarios like analyzing California Rim Fire remote sensing data. This minimizes false alarms, reduces unnecessary resource deployment, and enhances interpretable decision-making for fire suppression strategies. Complementing these, latency evaluates real-time query processing in the conversational UI, ensuring timely responses to user prompts on FLAME 3 UAV imagery for rapid evacuation planning. Throughput measures the orchestrator-based architecture’s capacity to handle concurrent data streams from different sources, optimizing agent collaboration during peak wildfire events. Finally, resource utilization informs efficient allocation across LMM and RAG pipelines, enabling edge–device deployment for sustainable, field-deployable management without sacrificing contextual reasoning on diverse datasets such as the National USFS Fire Occurrence Point.

### 4.3. Web Application with a Visual Question–Answer System

**Figure 5:**
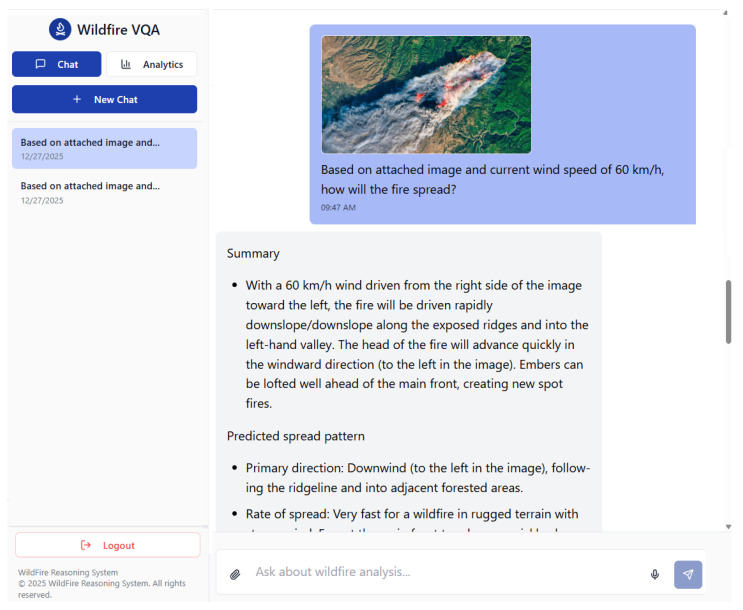
Conversational User Interface.

The VQA application was developed with the objective of enabling natural and intuitive interactions, akin to human conversation, as illustrated in Figure 5. The interface features a clean and uncluttered layout, incorporating a sidebar on the left for efficient navigation through previous conversations. The central screen focuses on the ongoing dialogue, thereby maintaining user attention on the interaction. Constructed as a contemporary web application utilizing React 18.3.1 and TypeScript 5.8.3, along with the shadcn/UI 0.9.5 component library, the system guarantees high performance, reliability, and cross-device compatibility. The system follows a three-tier architecture model built using Vite 5.4.19 for optimized compilation and TanStack Query 5.83.0 for efficient server-state synchronization. The presentation layer captures multimodal inputs and communicates via RESTful APIs with the backend application layer, where the Orchestrator Agent manages execution logic, supported by a data layer that leverages vector databases for high-speed RAG retrieval.

The core functionality resides in the bottom input bar, offering user-friendly query submission options. These include typing detailed questions, uploading up to five images for contextual enhancement, or voicing queries aloud. Voice inputs are accurately transcribed into text, facilitating hands-free operation and on the move usability. Beyond basic querying, the system encompasses essential functional modules, including secure authentication, session management for conversation history persistence, and a markdown-based rendering engine. To enhance readability, the VQA’s responses are delivered in a structured and formatted manner. This multimodal approach that integrates text and images significantly increases the efficiency of the system and user accessibility. Case studies with specific user queries and responses of different models are available at our website [41].

### 4.4. Comparative Analysis of LLM

The proposed MAS was evaluated using five distinct LMM backbones to assess the trade-off between reasoning capability and operational efficiency. To ensure strictly comparable results, an identical, fixed test set was employed across all backbone evaluations. No model-specific data re-sampling or dynamic splitting was performed, ensuring that the performance metrics in Table 7 reflect architectural differences rather than data variances.

**Figure 6:**
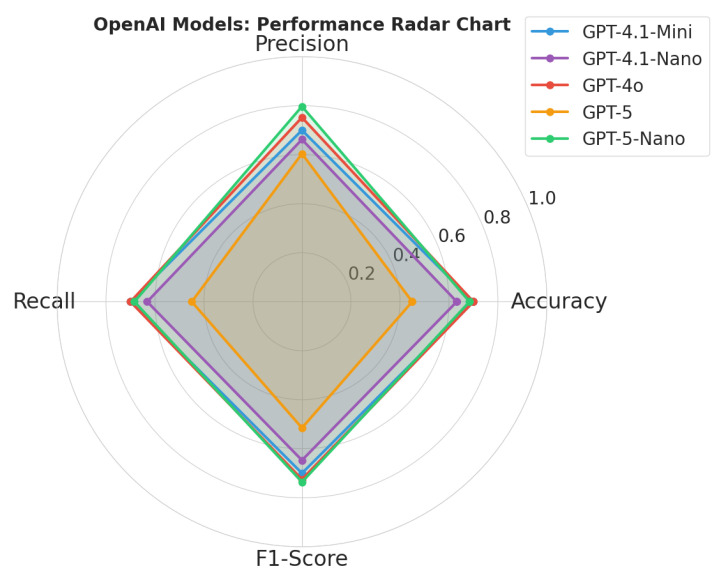
Radar graph for the performance of different models.

According to results shown in Table 7 and Figure 6, the GPT-4o model exhibits a symmetrical, balanced profile, maintaining equilibrium across Accuracy (0.700), Precision (0.752), and Recall (0.700). This distinct “diamond” shape indicates its suitability for general-purpose monitoring, where false negatives and false positives carry equal weight.

Conversely, GPT-5-Nano model demonstrates a skewed profile. While it achieves the highest system-wide Precision (0.797), it suffers from lower Recall (0.684). This asymmetry suggests the model adopts a conservative decision boundary, requiring high-confidence multimodal evidence before triggering an alert. In a wildfire context, this behavior is operationally advantageous for automated suppression systems, where false alarms incur high resource mobilization costs.

**Figure 7:**
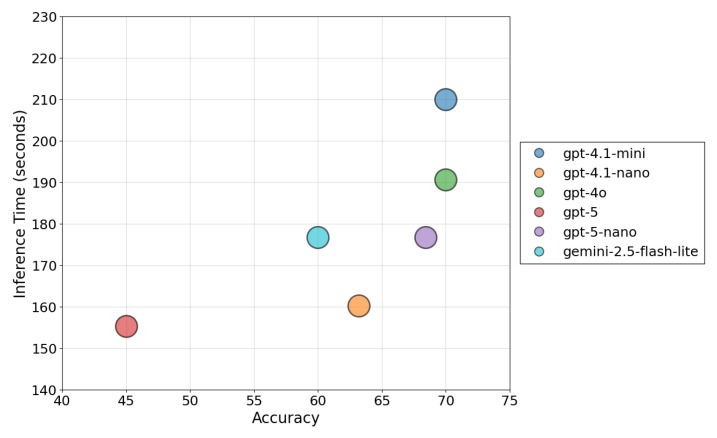
Accuracy vs. Inference Time.

Inference latency is a critical performance metric for real-time deployment. As shown in Figure 7 visualizes this efficiency frontier. The results highlight that GPT-4.1-Mini, despite its high accuracy, incurs a significant latency penalty (213.02 s), rendering it suboptimal for rapid-response scenarios. In contrast, GPT-5-Nano occupies the optimal region of the graph (in purple), delivering superior precision with a 17% reduction in inference time (176.70 s) compared to the GPT 4.1-Mini variant. This balance validates the selection of GPT-5-Nano as the primary reasoning engine for the Orchestrator Agent.

### 4.5. Ablation Study and Architectural Validation

**Figure 8:**
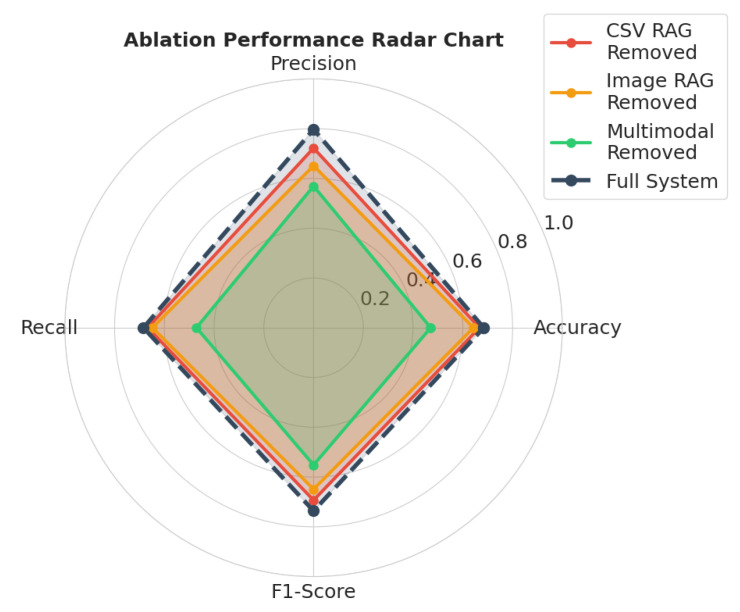
Visualization of ablation performance.

To quantify the dependency of the system on its multimodal components, an ablation study was performed by systematically disabling the CSV data retrieval, Image data retrieval, and multimodal RAG pipelines. Table 8 gives the quantitative performance observed across these configurations. The ablation analysis reveals a dependency on the multimodal RAG tool. As visualized in Figure 8, the removal of this component (green line) degrades performance by reducing accuracy from 0.684 to 0.471 and the F1-score from 0.736 to 0.515. This degradation confirms that the multimodal RAG pipeline functions as the main integration hub for the data acquisition agent. Without it, the system cannot effectively bridge the semantic gap between tabular data and unstructured visual inputs, leading to a breakdown in reasoning capabilities.

While the complete model incurs the highest inference latency (176.70 s), this cost is justified by the reliability gains. The “Multimodal Removed” configuration, while faster (116.94 s), yields unacceptable accuracy, validating the design choice to prioritize robust contextual integration over processing speed. Thus, the shrinkage of the “Multimodal Removed” polygon (green) reflects the system’s reliance on cross-modal synthesis for robust detection.

**Figure 9:**
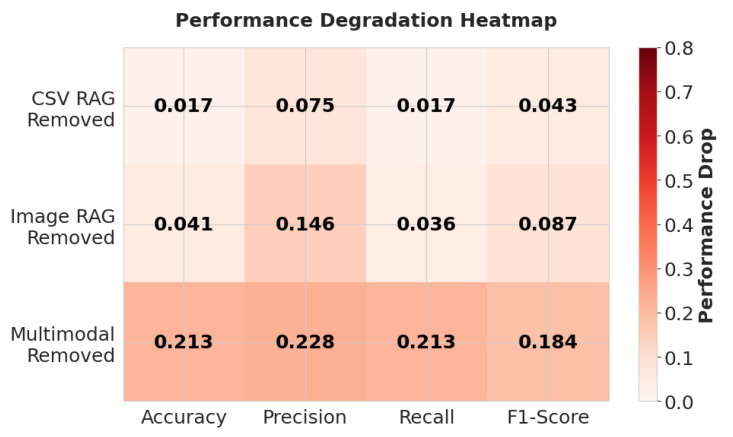
Performance degradation heatmap.

In contrast, independent removal of either CSV data retrieval tool or Image data retrieval tool resulted in only marginal performance drops (ΔAccuracy≈0.02−0.04). This phenomenon is highlighted in the degradation heatmap shown in Figure 9. Here, the darker cells indicate a severe impact on performance. The high intensity of the bottom row confirms that removing the multimodal RAG tool is the most detrimental ablation. The top rows show lighter intensities (lower performance drop) compared to the bottom row. This suggests that the architecture possesses inherent redundancy; when a single-modality tool is disabled, the data acquisition agent can leverage the multimodal RAG tool as a fallback mechanism to retrieve the missing context by preventing total system failure.

To quantitatively evaluate the factual accuracy of the system’s reasoning and ensure it does not hallucinate safety-critical advice, precision is utilized as a direct proxy for groundedness, validated against the labeled ground-truth datasets (FLAME 3 and Algerian Forest Fires). In wildfire management, the hazardous hallucination impacts primarily as false positives, where the system makes a positive, soundly reasoned warning in the absence of a hazard. Thus, a high precision score means that the system’s positive predictions are always backed by facts, and it does not hallucinate or make up its predictions. In GPT-5-Nano, the precision was scored at 0.797, indicating that the system’s reasoning was mostly linked to the positive examples rather than being counterfeit. The efficacy of the proposed architecture in the mitigation of hallucinations is proven through the results obtained in the ablation study, as depicted in Table 8. If the LMMs were using internal knowledge to reason (hallucination), the removal of the retrieval tools would have little effect on the reasoning process. However, the sharp drop in the F1-score, from 0.736 to 0.515, when the Multimodal RAG tool is removed, confirms the strong contextual dependency of the LMM. This thus establishes the causality between the retrieved information and the generated insight, thereby proving that the reasoning process is grounded in the provided data artifacts and not the hallucinated information.

However, this rigorous grounding imposes specific limitations that must be acknowledged. The system exhibits a conservative decision boundary, evidenced by a lower recall of 0.684, indicating a trade-off where the model prioritizes the elimination of false alarms over maximal sensitivity. The system requires strong cross-modal evidence, such as the presence of “smoke”, as signified visually and meteorologically, before it concludes with a “Fire” determination. In this way, it may overlook fires at their infancy or unclear cases such as wisps of smoke carried by strong winds. The system relies on corroboration via the RAG model. Therefore data availability is crucial. The system cannot deduce whether it is seeing a fire using a black box CNN model. Instead, it relies on available metadata such as wind speed, humidity, historical data, etc. The system may default to a negative determination via the safety rules it applies if it cannot get this data. This underlines the need for a strong data backbone to support the high precision claimed. Moreover, positional drift poses a challenge, particularly in high-velocity situations where the system’s inference latency of approximately 176 s can result in a significant lag, thus requiring a “look-ahead” capability to synchronize the location received by the system with the actual location of the fire front.

Qualitatively, the multi-agent system’s error profile is shaped by systems architectural emphasis on high-precision reasoning over raw sensitivity. The most observed error category is False Negative, which stems from the model’s conservative decision boundary (Recall: 0.684). In some scenarios characterized by ambiguous visual cues, such as low-density smoke dispersed by high winds or thermally masked heat signatures, the reasoning agent try to reject the alert unless it is strongly corroborated by cross-modal evidence, at which time it leads to missed detections. High precision (0.797) minimizes the hallucinations, and contextual misalignment errors can be occurred if the retrieved historical analogs are semantically close but meteorologically differ. This potentially leads to accurate hazard detection but suboptimal spread prediction vectors.

## 5. Discussion

### 5.1. Lessons Learned

This study addressed the limitations of siloed data processing in wildfire management through the development of an orchestrator-based MAS. It was demonstrated that by integrating LMMs with specialized RAG pipelines, agentic workflows can effectively synthesize heterogeneous data, ranging from satellite imagery to historical fire records into coherent, context-aware decision support.

The findings presented in this research directly addressed the formulated research questions. RQ1 (Multimodal Integration) is achieved by the successful deployment of the data acquisition agent. It confirmed that a hierarchical agentic framework can dynamically retrieve and normalize disparate data formats, specifically tabular, visual, and sensor data, without the need for manual preprocessing. RQ2 (Operational Advantages) is justifiable by the comparative performance of the system that highlighted the value of the “Reasoning Agent.” Unlike traditional DL models that function as “black boxes,” the proposed architecture provides text-based reasoning. This shift from opaque probability scores to explained decisions was identified as critical for establishing trust with human incident commanders.

### 5.2. Comparative Analysis with State-of-the-Art Models

To validate the efficacy of the agentic approach, the system was benchmarked against established baselines in wildfire detection and management. Table 9 provides a high-level comparison of functional capabilities.

It was observed that DL models like SmokeyNet [14] excel at a high-speed pattern recognition but lack contextual awareness. Similarly, Fuzzy Inference Systems [7] and WSN controllers [8] provide efficient static monitoring yet struggle to adapt to dynamic, unstructured queries. In contrast, the proposed orchestrator-based system, while incurring higher latency (176.70 s), introduces a critical layer of semantic reasoning absent in these baselines, achieving a precision of 0.797. This performance profile is defined here as ‘Strategic Real-Time’. Compared to the standard manual coordination cycle (approx. 30 min), this latency delivers substantial operational acceleration.

To prevent this computation window from undermining the physical validity of responses, we integrated a predictive look-ahead mechanism. Instead of optimizing for the state at data ingestion ($t_{0}$), the system requests wildfire spread predictions for a future time horizon ($t_{0}+\Delta t$), where Δt covers the inference latency. As a result, generated resource allocation plans target the anticipated wildfire state at execution time, preserving decision intelligence despite the strategic latency.

To analytically estimate the operational impact of the predictive look-ahead mechanism, we conducted a sensitivity analysis simulating ‘Positional Drift’, which can be defined as the spatial error induced by inference latency, across a spectrum of wind velocities (20–80 km/h). We compared our approach against a Standard Non-Predictive Baseline, defined as a detection system that reports coordinates based solely on the data ingestion timestamp ($t_{0}$), without compensating for the processing duration (Δt).

As detailed in Table 10, the system’s benchmarked average inference latency (Δt) of 176.70 s creates a significant spatial discrepancy that scales linearly with wind intensity. For instance, under a high-velocity benchmark of 60 km/h (ROS ≈ 100 m/min) typical of wind-sensitive shrublands, standard system latency results in a theoretical positional error of approximately 294.5 m (2.945min×100m/min). Table 10 demonstrates that across this entire operational range, integrating the $t_{0}+\Delta t$ look-ahead projection mitigates systematic drift, reducing the spatial error to the marginal stochastic variance of the model’s inference time.

**Figure 10:**
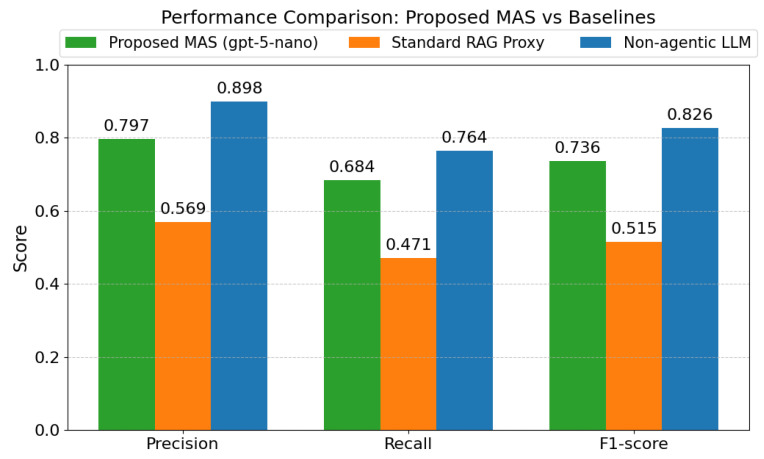
Performance of the Proposed MAS against Standard RAG and Non-agentic LLM baselines.

Figure 10 presents a performance comparison between the proposed MAS (utilizing GPT-5-Nano), a standard RAG Proxy, and a non-agentic LLM [14]. The results demonstrate that the orchestrator-based architecture significantly outperforms the standard RAG proxy, improving the F1-score from 0.515 to 0.736 and Precision from 0.569 to 0.797. Although the non-agentic model has a higher value of overall classification performance, including a precision of 0.898, it behaves like a ‘black box,’ with no specific cross-modal grounding applicable to disaster response tasks. Unlike the proposed MAS, where the outputs are validated by using the externally retrieved evidence, non-agentic approaches only use the parametric knowledge present in the model, resulting in a high chance of unverifiable hallucinations with no possible links to the original data.

Unlike generic agentic RAG systems that typically rely on open-ended ReAct loops and lossy image-to-text captioning, the proposed framework introduces a deterministically orchestrated architecture optimized for safety-critical environments. A key differentiator is the implementation of a decay-weighted routing policy ($\pi (q,C_{t})$), which mathematically forces task convergence to prevent the infinite retrieval loops often observed in standard autonomous agents. Furthermore, the system advances beyond standard multimodal RAG by utilizing a ‘lossless’ retrieval pipeline that injects raw Base64 visual artifacts directly into the LMMs context window, thereby preserving granular visual features that are discarded during the intermediate captioning steps of conventional frameworks. Finally, stochastic variability is strictly managed through a formalized context engineering tuple (p=(ρ,γ,τ,ϵ,ϕ)), which replaces loose natural language prompting with rigid state definitions to ensure the reproducibility required for disaster management.

We use the ‘Non-agentic LLM’ baseline to approximate the behaviour of black-box models like SmokeyNet [14]. While these models achieve superior precision (0.898) through strict visual pattern matching, they fail to distinguish benign events from genuine hazards. A prime example is a prescribed burn, where a black-box model simply detects ‘smoke’ and triggers an alert; our reasoning agent validates that visual input against active burn permits via the CSV RAG pipeline. Although this cross-verification yields a more conservative F1-score (0.736), it provides a critical operational advantage: effectively filtering false positives and contextualizing the event as ‘controlled’ rather than triggering the unnecessary mobilization typical of purely visual detection.

While recent studies have explored MAS for wildfire suppression, reliance is often placed on conceptual frameworks or simulations. For instance, Tavakol et al. [6] and Zadeh et al. [20] proposed swarm-based architectures for UAV coordination. While these approaches excel in coverage optimization, it was noted that they lack the cross-modal data synthesis capabilities of the orchestrator-based approach.

As detailed in Table 11, ML and DL models like SmokeyNet achieve higher raw performance metrics (e.g., Precision 89.84%) compared to the proposed system (Precision 79.7%). However, this disparity reflects the fundamental difference in operational scope. SmokeyNet is a specialized ‘narrow AI’ optimized solely for binary visual classification (Smoke vs. No Smoke) within a closed dataset. In contrast, the orchestrator-based MAS is designed for dynamic, open-world reasoning, integrating heterogeneous inputs such as wind speed, historical burn records, and thermal imagery.

In government decision-support scenarios, the marginal reduction in raw classification precision represents a necessary trade-off for achieving operational explainability. While black-box models may excel in pixel-level detection, they lack the semantic capacity to explain the cause or context of a fire. Our proposed system prioritizes the validity of final decisions such as evacuation planning, over the raw sensitivity of initial detection, ensuring incident commanders receive actionable intelligence rather than isolated alerts.

However, the tendency of LLM to hallucinate and the system’s overall accuracy being below 0.8 necessitate the need for the system to be deployed in an operationally rigorous manner. In the real-world scenario of natural disasters, the framework has the tendency to function more like a human-in-the-loop Decision Support System (DSS) rather than an autonomous system for decision-making, in which explainability becomes the key safety aspect of the system’s functioning. Unlike high-accuracy ‘black box’ models that may confidently misclassify benign events (false positives) without justification, the proposed system exposes its reasoning chain, allowing incident commanders to audit the logic, for instance, verifying if a fire spread prediction is physically consistent with the retrieved data. This transparency allows human experts to detect and discount hallucinations before taking action. To further enhance system reliability beyond the current human-in-the-loop paradigm, future work will integrate technical mitigations to address hallucinations. Implementing self-consistency mechanisms, where the model generates multiple reasoning chains to identify a consensus output, and filtering out stochastic errors can increase the power of the network. Integrating uncertainty quantification metrics that assign confidence scores to predictions, automatically flagging low-certainty outputs for mandatory manual verification, is another solution.

### 5.3. Challenges and Future Works

Despite these contributions, the proposed model is bottleneck with inference latency. As mentioned in the results, the reliance on LMMs introduces response delays (approximately 176 s for GPT-5-Nano), which may be prohibitive for real-time suppression schemes, where sub-second reaction times are required. Furthermore, the computational cost of continuous RAG retrieval presents a barrier to deployment on resource-constrained edge devices. To address these challenges, the proposed multimodal agentic AI framework with RAG-based VQA offers substantial potential for extension, building on its current strengths in synthesizing visual, textual, and geospatial data for wildfire insights while addressing limitations in computational efficiency and multimodal coverage.

This framework can be extended to incorporate an audio RAG module to process forest acoustic data, enabling classification of fire-specific sounds such as crackling flames and animal distress calls [44]. This would leverage audio-language models integrated with agentic workflows, allowing autonomous agents to triage auditory alerts in real time and fuse them with visual VQA outputs for richer situational awareness. Moreover, real-time multimodal integration will be pursued by coupling the system with streaming data sources such as satellite feeds, IoT sensors, and drone imagery [45]. Similarly, land cover and land use fusion will incorporate high-resolution datasets into the decision-making pipeline, empowering agents to assess vulnerability of infrastructure and biodiversity hotspots [13]. Consequently, agentic orchestration frameworks could enable dynamic risk analysis, where specialized agents negotiate insights such as smoke plume prediction via diffusion models, evacuation routing, and update the RAG knowledge base on-the-fly, enhancing responsiveness in operational wildfire centers. Additionally, a judge agent can be implemented to assess the outputs and decisions of other agents to ensure accuracy, reliability, and alignment with real-time priorities, thereby minimizing errors in critical scenarios [46].

Additionally, optimization strategies for scalability, including model distillation, quantized embeddings, and federated learning across edge devices in disaster-prone regions, can be designed and utilized to tackle computational overhead from pretrained models and vector databases [47,48,49]. This ensures low-latency inference without sacrificing accuracy, critical for large-scale deployment.

Importantly, chain-of-thought prompting is a crucial aspect, as it decomposes intricate reasoning processes into sequential intermediate steps, significantly boosting accuracy, interpretability, and performance on multimodal challenges [45,50].

Generally, black-box models often obscure the reasoning of the solution, making it difficult for stakeholders to validate outputs against ground realities or regulatory standards. By providing interpretable insights such as attention maps highlighting key image features or reasoning chains, XAI enhances model accountability, facilitates error debugging, and supports interdisciplinary collaboration among scientists, policymakers, and local communities through VQA systems [27,28,29]. Ultimately, this transparency empowers more reliable, ethical applications in high-stakes scenarios like rapid disaster response, where opaque predictions could lead to misguided interventions. Further, post-disaster assessment can integrate AI-driven MAS to enable real-time, predictive evaluations of burn severity, erosion risks, and recovery needs, enhancing proactive rehabilitation efforts [51].

Finally, the framework will evolve toward a standardized, interoperable architecture, facilitating multi-agenct collaboration. Extensions to other disasters, including floods via hydroacoustic RAG and United Nation’s sustainable development goals (SDG), particularly SDG 13 (climate action) and SDG 15 (life on land), will promote forest preservation as carbon sinks, climate resilience, and reduced deforestation through proactive, non-technical VQA access for responders [21]. These advancements position the system as a versatile tool for agentic AI in environmental disaster management.

## 6. Conclusions

This paper proposed a novel orchestrated multi-agent framework designed to transform the landscape of wildfire management through context-aware, multimodal reasoning. By integrating LMMs with specialized RAG pipelines, the proposed system successfully bridged the gap between heterogeneous data streams, including satellite imagery, meteorological tabular records, and ground-based footage, enabling a human-centric VQA interface. Experimental validation across diverse geographical datasets demonstrated a precision of 0.797 and an F1-score of 0.736. While inference latency remains a challenge for real-time suppression, the modularity of the architecture provides a robust foundation for future extensions. Finally, this work aligns with global sustainability goals by providing decision-makers with a proactive, scalable, and adaptive tool for ecosystem preservation and disaster resilience.

## Figures and Tables

**Table 1:** Architectural contrast between standard Agentic RAG paradigms and the proposed framework.

Feature	Standard Agentic RAG	Proposed Framework (Ours)	Architectural Advantage
Orchestration Logic	Recursive ReAct Loops: Relies on open-ended ‘Reason + Act’ cycles that often loop indefinitely or hallucinate tools in complex scenarios.	Decay-Weighted Routing: Implements a policy function $\pi (q,C_{t})$ with a decay factor $\lambda_{decay}$ that mathematically forces task convergence.	Prevents infinite loops and ensures deterministic latency for safety-critical response.
Multimodal Data	Intermediate Captioning: Converts images to text descriptions before processing, causing loss of granular visual details (e.g., smoke density).	Lossless Artifact Injection: Retrieves and injects raw Base64 visual artifacts directly into the LMM context window.	Preserves forensic visual fidelity required for distinguishing similar hazards (e.g., cloud vs. smoke).
Context Management	Unstructured Logs: Appends raw conversation history to the prompt, leading to context drift and unauthorized tool usage.	Formalized State Tuples: Uses a rigid tuple structure p=(ρ,γ,τ,ϵ,ϕ) to strictly define role boundaries and constraints.	Guarantees reproducibility and prevents agents from acting outside safety guardrails.

**Table 2:** Overview of Related Studies on Wildfire Screening.

Study	Description	Approach				Region
		Rule-Based	Fuzzy Logic	AI	Other	
SmokeyNet (2022) [14]	Multimodal smoke detection	–	–	CNN, LSTM, ViT	–	USA (HPWREN sites)
UAV Swarms for WER (2025) [6]	Autonomous UAV wildfire suppression	Simple rules	–	MAS, Swarm Robotics	–	United Kingdom
High-Level MAS with DRL (2025) [20]	MAS with DRL for fire tracking	–	–	MAS, Deep RL	–	Global
Heterogeneous MAS (2025) [18]	UAV/ground robot monitoring	–	–	MAS	–	–
MARL-based Systems (2020) [19]	Swarm ocean monitoring	–	–	Multi-Agent RL	–	Bedok Reservoir, Singapore
WildfireGPT (2025) [15]	RAG-based LLM decision support	–	–	MAS, LLM, RAG	–	United States
Fuzzy Fire Mapping (2024) [7]	Fire susceptibility mapping	–	Fuzzy Inference System	–	–	Brazil (Rondônia)
WSN Fire Controller (2018) [8]	IoT/WSN Fire Controller	–	Dynamic Fuzzy Logic	–	IoT, WSN	Spain
Edge-UAV System (2018) [33]	UAV Early Detection	–	–	–	Edge Computing	European South Region

**Table 3:** Comparison of Existing Systems.

Study	MAS Architecture	UAV/Drone Swarms	Multi Modal Fusion	VQA	Context Awareness	Historical Data	RAG	MAS Orchestration	Explainable Decisions
SmokeyNet [14]	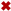	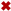	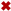	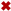	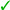	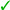	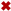	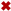	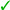
UAV Swarms for WER [6]	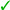	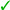	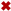	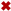	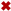	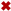	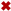	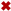	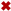
High-Level MAS with DRL [20]	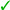	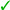	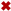	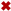	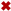	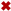	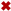	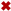	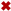
Heterogeneous MAS [18]	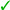	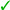	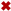	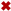	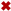	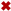	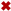	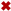	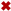
WildfireGPT [15]	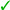	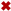	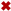	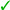	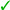	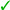	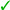	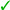	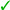
MARL-based Systems [19]	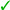	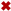	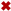	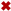	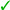	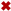	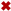	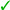	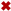
Fuzzy Fire Mapping [7]	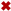	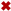	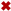	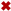	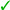	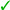	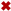	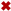	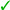
WSN Fire Controller [8]	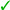	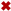	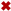	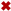	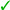	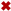	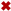	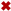	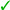
Edge-UAV System [33]	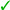	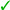	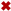	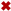	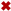	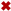	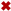	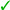	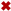
Proposed VQA-MAS System (Ours)	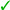	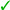	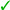	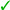	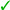	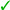	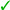	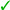	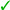

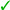
 Satisfies; 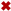 Does Not Satisfy.

**Table 4:** Dataset features and reasons for using them.

Dataset	Type	Description	Features
Algerian Forest Fires [34]	Tabular	244 instances from Bejaia (northeast) and Sidi Bel-Abbes (northwest) regions in Algeria, 122 per region.	Contains meteorological data for in CSV format, indexed in the vector DB, and retrieved from the CSV Retrieval pipeline
Remote Sensing Data Before and After California Rim and King Forest Fires, 2010–2015 [36]	Satellite and thermal images	High-resolution surface reflectance, thermal imagery, burn severity metrics, and LiDAR-derived structural measures from Sierra Nevada Mountains, California, USA, collected before/after 2013 Rim and 2014 King fires.	Provides high-resolution multi-spectral and thermal imagery, indexed in vector DB and utilized in image retrieval pipeline.
National USFS Fire Occurrence Point [37]	Tabular	Ignition points for USFS wildland fires, maintained at Forest/District level to track occurrence and origin.	Provides historical US wildfire data for VQA to understand fire patterns and geographical risks.
FLAME 3 - Radiometric Thermal UAV Imagery for Wildfire Management [35]	UAV images/thermal	622 image quartets labeled Fire and 116 labeled No Fire from the surrounding forestry of the prescribed burn plot.	Gives RGB-thermal image pairs for multimodal fusion algorithms.

**Table 5:** Orchestrator Decision Logic Matrix.

Query Condition	Dominant Weight Factor	Selected Agent	Execution Action Order
System Initialization (e.g., “New User Query *q* Received”)	Policy Initialization ($\pi (q,C_{0})$) Orchestrator evaluates: is β (Data Need) or α (Intent Match) the priority?	Orchestrator Agent	1. Parse semantic intent of query *q*. 2. Instantiate dynamic weights (α,β). 3. Determine initial $a_{target}$. 4. Route control to the selected agent (typically Data Acquisition for complex queries).
Data Retrieval (External Context Required)	High Data Need (β>α)	Data Acquisition Agent	1. Execute RAG pipelines. 2. Update global context ($C_{t}$). 3. Data validation: If data is null, re-invoke Data Acquisition Agent immediately. 4. Once data is sufficient, decay β ($\beta \leftarrow \beta \cdot \lambda_{decay}$) to shift control.
Context Reasoning Sufficient Data Available	High Intent Match (α>β) Prioritizes logic synthesis and answer generation.	Reasoning Agent	1. Ingest grounded context ($C_{t}$). 2. Perform Chain-of-Thought analysis. 3. Draft response (*R*). 4. Submit *R* to Orchestrator Agent.
Ambiguous/Conflict (e.g., Conflicting visual vs. textual data)	Recursive Correction Orchestrator detects low confidence or format violation.	Recursive Loop	1. Trigger Supervisor–Worker Protocol 2. Re-invoke subordinate agent with refined constraints 3. Filter Hallucinations

**Table 6:** A comparison between Task-based and Orchestrator-based approaches.

Ability	Task-Based Approach	Orchestrator-Based Approach
Dynamic task planning	No	Yes
No of crew needed	Single	Multiple
Agent invocation	Sequential, fixed	Flexible, orchestrated
Maintainability	Direct, less flexible	Modular, more maintainable
Task execution flow	Crew executes tasks	Orchestrator agent manages flow
Use case suitability	Simple, linear flows	Complex, adaptive workflows

**Table 7:** Quantitative performance comparison of OpenAI and Gemini models across wildfire detection tasks.

Model	Accuracy	Precision	Recall	F1-Score	Inference Time (s)
GPT-4.1-Mini	0.700	0.700	0.700	0.700	213.02
GPT-4.1-Nano	0.632	0.662	0.632	0.647	160.21
GPT-4o	0.700	0.752	0.700	0.725	190.57
GPT-5	0.450	0.604	0.450	0.515	155.25
GPT-5-Nano	0.684	0.797	0.684	0.736	176.70
Gemini-2.5-Flash-Lite	0.600	0.662	0.600	0.629	176.70

**Table 8:** Ablation results with the selected GPT-5-Nano LMM.

Ablation Type	Accuracy	Precision	Recall	F1-Score	Inference Time (s)
Without CSV Data Retrieval tool (Only Image Data Retrieval tool and Multimodal RAG tool)	0.667	0.722	0.667	0.693	94.50
Without Image Data Retrieval tool (Only CSV Data Retrieval tool and Multimodal RAG tool)	0.643	0.651	0.648	0.649	107.96
Without Multimodal RAG tool (Only Image Data Retrieval tool and CSV Data Retrieval tool)	0.471	0.569	0.471	0.515	116.94
With CSV Data Retrieval tool, Image Data Retrieval tool and Multimodal RAG tool	0.684	0.797	0.684	0.736	176.70

**Table 9:** Functional comparison of the proposed MAS against existing approaches.

Approach	Performance	Detection Speed	Monitoring Capability	Management Support	Scalability
WSN Fire Controller (2018) [8]	N/A (real-time alerts)	Real-time (WSN)	Sensor-based variables	Risk alerts	High (multi-hop routing)
Edge-UAV System (2018) [33]	N/A (qualitative efficient management of CPU/RAM, battery life, and network resources based on initial experiments)	Real-time (edge/fog)	UAV detection	Resource allocation	High (hierarchical)
MARL-based Systems (2020) [19]	Learning convergence	Real-time inference	Spatial coverage	Monitoring	Medium (dataset dependent)
SmokeyNet (2022) [14]	Accuracy: 83.49%	Real-time inference	Image	Smoke Detection	Medium (dataset dependent)
Fuzzy Fire Mapping (2024) [7]	AUC 0.879	N/A (static mapping)	Susceptibility via GIS	Prevention actions	Medium (climate-sensitive)
UAV Swarms for WER (2025) [6]	N/A (conceptual)	N/A (conceptual)	Real-time via UAVs	Evacuation/ Suppression	High (swarm-based)
High-Level MAS with DRL (2025) [20]	N/A (conceptual)	Real-time (DRL algorithms)	UAV/IoT tracking	DSS for decision-making	Medium (integrated data)
Heterogeneous MAS (2025) [18]	Finite-time convergence (simulations)	Finite-time tracking	Cooperative air-ground	Fault-tolerant tracking	High (heterogeneous agents)
WildfireGPT (2025) [15]	Correctness: 97.73% (case studies)	Real-time inference (LLM-based)	Data synthesis (climate projections/literature)	Risk insights/ decision-making	High (LLM scalable)
Proposed MAS	Precision 0.797, F1-score 0.736	Low latency (orchestration)	Multimodal synthesis	Context-Aware Reasoning	High (agentic scalability)

**Table 10:** Sensitivity analysis of Positional Drift: Comparing theoretical spatial error between a Standard Non-Predictive Baseline and the proposed look-ahead mechanism across a spectrum of wind intensities.

Wind Speed (km/h)	Est. Rate of Spread (ROS) [m/min] ^1^	Inference Latency (Δt) [sec]	Spatial Error (Baseline) [m] ^2^	Spatial Error (Ours) [m]
20	≈33	176.70	97.1	Residual Variance
40	≈66	176.70	194.3	Residual Variance
60 (Benchmark)	≈100	176.70	294.5	Residual Variance
80	≈133	176.70	391.6	Residual Variance

^1^ ROS values are parameterized using a conservative wind-coupling ratio ($ROS\approx 0.1\cdot V_{wind}$) to simulate high-intensity propagation. This “10% Rule of Thumb” is a standard operational heuristic for shrublands [42], consistent with the wind-driven physics described in the Rothermel model (p. 14, [43]). ^2^ Calculated as linear displacement (d=ROS×Δt) for a system without temporal compensation.

**Table 11:** Architectural comparison highlighting the shift from static inference in static models to dynamic, context-aware reasoning in the proposed orchestrator-based MAS.

Approach	Architecture	Data Handling Strategy	Reasoning Process	Performance Metrics
WSN Fire Controller (2018) [8]	WSN with fuzzy controller	Sensor (meteorological/gases)	Fuzzy logic for alerts	N/A (real-time alerts)
Edge-UAV System (2018) [33]	Edge/fog/cloud hierarchy	UAV sensor data	Dynamic allocation	N/A (qualitative efficient management of CPU/RAM, battery life, and network resources based on initial experiments)
MARL-based Systems (2020) [19]	MARL networks	Continuous 2D position states and movement actions	Reinforcement learning with centralized training	Convergence in 5000 episodes (CR-MARL)
SmokeyNet (2022) [14]	Single-model CNNs	Static sequential image frames from fixed cameras	Fixed inference/Binary classification	Precision: 89.84%, Recall: 76.45%, F1-score: 82.59%, Accuracy: 83.49%
Fuzzy Fire Mapping (2024) [7]	Fuzzy inference with GIS	Remote sensing (temp/rainfall)	Rule-based susceptibility	AUC 0.879
UAV Swarms for WER (2025) [6]	Systems engineering with swarms	Multimodal (sensors/UAVs)	Collaborative self organization	N/A (conceptual)
High-Level MAS with DRL (2025) [20]	Hierarchical MAS	Integrated historical/real-time	DRL for tracking/estimation	N/A (conceptual)
Heterogeneous MAS (2025) [18]	Fault tolerant formation control	Sensor inputs for tracking	FO-NFTSM/HOSMO	Finite-time convergence
WildfireGPT (2025) [15]	LLM Agent with RAG framework	Multi-modal data sources	Multi-round conversational reasoning	Correctness: 97.73%, Relevance: 98.20%, Entailment: 93.75%, Accessibility: 95.49%
Proposed Orchestrator- Based MAS (Ours)	Orchestrator based with LMM/RAG	Dynamic multimodal (text/image)	Context-aware via agents	Precision 0.797, F1-score 0.736 (Section 5, Table 7)

## Data Availability

Publicly available datasets were analyzed in this study. This data can be found here: Algerian Forest Fires Dataset [34], California Rim and King Forest Fires data [36], National USFS Fire Occurrence Point data [37], and the FLAME 3 Dataset [35].

## Appendix A. Multimodal Wildfire Reasoning and Response Tasks

The “Multimodal Wildfire Reasoning and Response” task formulated in Section 4.1 is composed of three distinct sub-tasks designed to simulate the decision-making pipeline of an incident commander. Table A1 details the input, objective, and output for each sub-task.

**Table A1:** Definitions of the sub-tasks comprising the Multimodal Wildfire Reasoning and Response evaluation.

Task ID	Task Name	Definition and Scope
Task 1	Hazard Identification	Objective: Detect the presence of active fire threats by cross-referencing visual artifacts with meteorological data.
	(Detection)	Input: Satellite/UAV imagery + Wind/Temperature data.
		Output: Binary Classification (Fire/No Fire) and confidence score.
Task 2	Spread Prediction	Objective: Forecast the future direction and rate of spread (ROS) of the identified fire front.
		Input: Wind direction, wind speed, and terrain features.
		Output: Directional vector and estimated ROS (e.g., “rapid downslope expansion”).
Task 3	Response Planning	Objective: Formulate particularized mitigatory action plans based on the synthesized threat assessment.
		Input: Synthesized outputs from Task 1 and Task 2.
		Output: Actionable recommendations (e.g., “initiate evacuation in Sector 4”).

## Appendix B. Mathematical Notations

The following table summarizes the mathematical notations, variable definitions, and processing functions used throughout the proposed Multimodal RAG pipeline.

**Table A2:** Summary of mathematical notations and definitions used in the framework.

Category	Notation	Description
Data Domains	$D_{tab}$	$\left\{r_{1},r_{2},\ldots ,r_{n}\right\}$, where each $r_{i}\in R^{d}$ represents a meteorological record with *d* features (temperature, humidity, wind speed, etc.).
	$D_{img}$	$\left\{I_{1},I_{2},\ldots ,I_{m}\right\}$, where each $I_{j}\in R^{H\times W\times C}$ denotes satellite or aerial imagery with height *H*, width *W*, and *C* color channels.
Hyperparameters	$\tau_{miss}$	Missing value threshold for row removal (default: 0.40). Range ∈[0,1].
	$\theta_{iqr}$	Interquartile Range (IQR) multiplier for outlier detection (default: 1.5). $\theta_{iqr}\in R^{+}$.
Embedding Models	${\mathrm{Model}}_{BAAI}$	Text embedding function ($T\to R^{768}$) using BAAI/bge-base-en-v1.5, mapping text strings to 768-dimensional vectors.
	${\mathrm{Model}}_{CLIP}$	Multimodal embedding function ($I\cup T\to R^{512}$) using OpenAI CLIP, mapping images or text to 512-dimensional vectors.
Vector Indices	${\mathrm{Index}}_{tab}$	Vector database storing tabular embeddings $\left\{v_{i}\right\}$ with associated metadata.
	${\mathrm{Index}}_{img}$	Vector database storing visual embeddings $\left\{u_{j}\right\}$ with associated metadata.
Retrieval Params	*k*	Top-k retrieval count ($k\in N^{+}$), specifying the number of most similar items to retrieve per search query.
Context Variables	*q*	User query in natural language (q∈T).
	$q_{vec}$	Text embedding of query *q* for tabular search ($q_{vec}\in R^{768}$).
	$q_{clip}$	CLIP embedding of query *q* for visual search ($q_{clip}\in R^{512}$).
	$C_{tab}$	Retrieved tabular context (top-k meteorological records).
	$C_{img}$	Retrieved visual context (top-k similar images).
	$C_{aug}$	Augmented prompt combining *q*, $C_{tab}$, and $C_{img}$.
	*A*	Final generated answer.
Functions	$\mathrm{MissingRatio}(r_{i})$	Computes fraction of missing values in row $r_{i}$. Output ∈[0,1].
	$\mathrm{Impute}(r_{i})$	Fills missing values using statistical methods (mean for numerical, mode for categorical).
	RemoveOutliers	Removes values outside $\left[Q_{1}-\theta \cdot \mathrm{IQR},Q_{3}+\theta \cdot \mathrm{IQR}\right]$.
	$\mathrm{Normalize}(r_{i})$	Applies Z-score normalization: $z=\frac{x-\mu }{\sigma }$.
	SerializeToText	Converts structured row data to natural language text $t_{i}\in T$.
	IntegrityCheck	Validates image file integrity. Returns {true,false}.
	$\mathrm{IsDuplicate}(I_{j})$	Detects duplicate images via perceptual hashing. Returns {true,false}.
	$\mathrm{Resize}(I_{j})$	Normalizes image resolution to standard dimensions.
	SearchKNN(v,k)	Retrieves *k* nearest neighbors to vector *v* using cosine similarity.
	PromptAugment	Constructs structured prompt template combining query *q* and retrieved contexts $C_{tab},C_{img}$.
	${\mathrm{Model}}_{Gen}\left(C_{aug}\right)$	Large Multimodal Model that generates final reasoning *A* from augmented context.
